# Luteolin and Chrysin Could Prevent *E. coli* Lipopolysaccharide-Ochratoxin A Combination-Caused Inflammation and Oxidative Stress in In Vitro Porcine Intestinal Model

**DOI:** 10.3390/ani12202747

**Published:** 2022-10-13

**Authors:** Annelie Wohlert, Nikolett Palkovicsné Pézsa, Alma Virág Móritz, Ákos Jerzsele, Orsolya Farkas, Erzsébet Pászti-Gere

**Affiliations:** Department of Pharmacology and Toxicology, University of Veterinary Medicine, István utca 2, 1078 Budapest, Hungary

**Keywords:** ochratoxin, IPEC-J2 cells, oxidative stress, interleukins, flavonoids, lipopolysaccharide, cytotoxicity

## Abstract

**Simple Summary:**

Environmental stressors, such as mycotoxins and enteropathogenic bacteria, can exert a detrimental effect on the gastrointestinal tract of the swine on contaminated corn-based feed which leads to the development of retarded growth; weight loss, thus, contributes greatly to lowered meat productivity. Plant-derived compounds such as flavonoid-type chrysin and luteolin were investigated in porcine enterocytes to evaluate their beneficial properties in gut damage. Toxic effects of ochratoxin A mycotoxin and bacterial outer membrane component, lipopolysaccharide could not be alleviated by the applied flavonoids even in combinations. However, luteolin and chrysin could prevent the excessive inflammatory responses by lowering cytokines’ production elicited by mycotoxin and bacterial toxin. Due to their antioxidant properties, these flavonoids were effective in compensating redox homeostasis tipped by combined exposure of ochratoxin A and inflammatory lipopolysaccharide. The porcine intestinal epithelial cell model applied by us was proven to be suitable for studying the harmful effects of the toxins and at the same time to evaluate the protective effects of flavonoids without living animal sacrifices. This study also suggests that mycotoxin-weakened intestinal function might result in the development of enteropathogen-caused secondary infections.

**Abstract:**

Ochratoxin A (OTA) and lipopolysaccharide (LPS) intake can cause gastrointestinal disorders. Polyphenolic chrysin (CHR) and luteolin (LUT) display anti-inflammatory and antioxidant properties. Porcine intestinal epithelial (jejunal) IPEC-J2 cells were treated with OTA (1 µM, 5 µM and 20 µM), *E. coli* LPS (10 µg/mL), CHR (1 µM) and LUT (8.7 µM) alone and in their combinations. Cell viabilities (MTS assay) and extracellular (EC) hydrogen-peroxide (H_2_O_2_) production (Amplex red method) were evaluated. Intracellular (IC) reactive oxygen species (ROS) were assessed using a 2′-7′dichlorodihydrofluorescein diacetate (DCFH-DA) procedure. ELISA assay was used to evaluate IL-6 and IL-8 secretion. OTA decreased cell viabilities (*p <* 0.001) which could not be alleviated by LUT or CHR (*p >* 0.05); however, EC H_2_O_2_ production was successfully suppressed by LUT in IPEC-J2 cells (*p <* 0.001). OTA with LPS elevated the IC ROS which was counteracted by CHR and LUT (*p <* 0.001). IL-6 and IL-8 secretion was elevated by LPS + OTA (*p <* 0.001) which could be inhibited by LUT (*p <* 0.01 for IL-6; *p <* 0.001 for IL-8). Based on our results, CHR and LUT exerted beneficial effects on IC ROS levels and on cytokine secretion (LUT) in vitro; thus, they might be used as dietary and feed supplements to avoid OTA- and LPS-related health risks.

## 1. Introduction

The post-weaning period is critical in large-scale piglet rearing. The weakened immune system makes piglets more susceptible to diseases caused by environmental facultative pathogens [1]. Intestinal inflammation reduces the growth rate of the animals and increases the mortality rate within the herd [2,3]. *Escherichia coli* (*E. coli*) strains can develop different pathologies in pigs depending on their virulence and the age and resistance of the animals. In pig herds, diarrheal disease caused by enterotoxigenic *E. coli* has high morbidity and can be fatal [4]. Verotoxin-producing *E. coli* strains cause enterotoxaemia, vascular damage, and sudden death in piglets after weaning, rarely in older piglets, especially on small farms. Enterohemorrhagic *E. coli* strains are of importance in foodborne *E. coli*-associated infections [5].

Intestinal infections in pigs are often treated with antibiotics, but the inappropriate use of these agents leads to the development of resistant bacterial strains, which renders therapy ineffective and may also pose a potential risk to humans consuming pork meat [6]. Therefore, there is a growing demand to find alternative substances for substitution or supplementation of antibiotic therapy.

**Figure 1:**
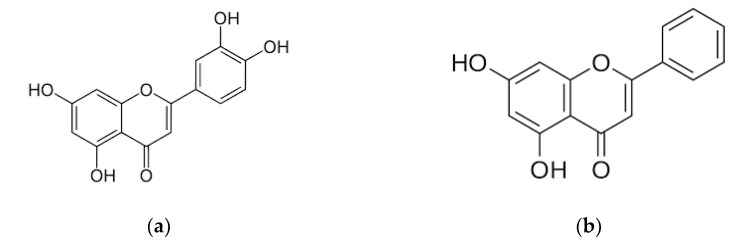
Chemical structures of (**a**) luteolin and (**b**) chrysin.

Flavonoids are secondary metabolites found in a wide variety of plants, mainly accumulated in vegetables, fruits and herbs [7]. Luteolin (LUT, 3′, 4′, 5, 7-tetrahydroxyflavone, Figure 1a) and chrysin (CHR, 5, 7-dihydroxyflavone, Figure 1b) have been reported to be an effective antioxidant, anti-inflammatory and antitumor adjuvant compounds [8,9,10]. Studying the effects of flavonoids on animal or human health is complicated by the fact that organisms may often be exposed to more than one harmful factor such as the presence of pathogens and various manifestations of environmental stress.

**Figure 2:**
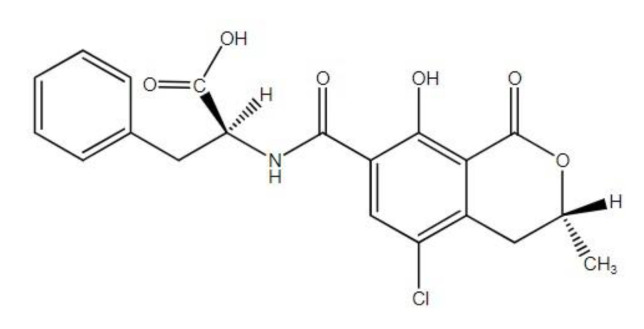
Chemical structures of ochratoxin A.

Among others, the presence of mycotoxins in feed can affect the susceptibility of animals to various infections [11]. Ochratoxin A (OTA, Figure 2) is a toxin derived from *Aspergillus* and *Penicillium* spp. and they are found mostly in the food chain and the feedstuff of animals. OTA is proven to be nephrotoxic, teratogenic, neurotoxic as well as immunotoxic and possibly carcinogenic to humans [12,13,14]. Consumption of contaminated pork meat-related products may lead to chronic toxicosis with OTA in humans. OTA levels were found to be significantly elevated in the blood serum and kidneys of porcine [15]. In addition, OTA is proven to have much longer half-lives in the blood of humans and pigs in comparison to any other animal species, which indicates a higher sensitivity of these species to OTA [12].

OTA could influence the cell viability of tumorigenic human colorectal adenocarcinoma CaCo-2/HT29-MTX cells [16]. A total of 48 h of treatment of cells with aflatoxin M1 or OTA (at 0.05 and 4 µg/mL) or with their combination caused significant cell death in human fetal FHs 74 intestinal cells [17]. OTA appeared to affect porcine intestinal jejunal epithelial cells (IPEC-J2) cell viability using both 3-(4,5-dimethylthiazol-2-yl)-2,5-diphenyltetrazolium bromide (MTT) assay and lactate dehydrogenase (LDH) method, and it also caused barrier dysfunction such as damage to the integrity of IPEC-J2 monolayers [18]. It was also found that OTA did not increase pro-inflammatory interleukin (IL)-8 secretion in Caco-2 cells directly; however, it could facilitate the impact of IL-1β-induced cytokine production in lower concentrations and it enhanced transepithelial passage of non-invasive commensal *E. coli* [19].

IPEC-J2 cell as an in vitro reliable model for swine intestine is a non-tumorigenic cell line of neonatal porcine small intestinal epithelial origin, which was isolated from the jejunum of newborn pigs, even before colostrum intake. The cells are characterized by a rapid proliferation and colonization ability which makes them appropriate for in vitro investigation of crosstalk between xenobiotics such as lipopolysaccharide (LPS) [20,21,22,23,24,25,26] and mycotoxins such as OTA [14,18,27,28] and swine enterocytes.

In our study, the cytotoxicity of individually applied and co-administered OTA and *E. coli* LPS and the effects of LUT and CHR were investigated by measuring cell viability rates in IPEC-J2 cells. In addition, markers of oxidative stress, such as extracellular (EC) hydrogen-peroxide (H_2_O_2_) production and intracellular (IC) reactive oxygen species (ROS) levels, were monitored after 24 h of exposure of IPEC-J2 cells to OTA or LPS alone or to their combinations either in the presence or in the absence of LUT and CHR. Changes in the secretion of proinflammatory cytokines such as IL-6 and IL-8 were also observed upon LPS/OTA treatment. Furthermore, potential beneficial effects of LUT and CHR were also elucidated in the restoration of upregulated IL-6 and IL-8 production and tipped redox balance of IPEC-J2 cells.

## 2. Materials and Methods

### 2.1. Reagents

Chemicals used in this study such as LUT, OTA, CHR, LPS solution originated from *E. coli* O111:B4 and porcine IL-6 and IL-8 enzyme-linked immunosorbent assay (ELISA) kits were obtained from Merck (Darmstadt, Germany). Amplex red hydrogen peroxide assay kit was purchased from Invitrogen (Thermo Fisher Scientific, Waltham, MA, USA) and CellTiter96 Aqueous One Solution reagent was from Promega (Madison, WI, USA).

### 2.2. Cells and Culturing Conditions

The cell line used for the experiments was the IPEC-J2 from the jejunum of the swine. The IPEC-J2 epithelial cell line was a kind gift from Dr. Jody Gookin (Department of Clinical Sciences, College of Veterinary Medicine, North Carolina State University, Raleigh, NC, USA). The cell monolayers were maintained in 75 cm^2^ cell culture flasks with filtered caps (Orange Scientific, Braine-l’Alleud, Belgium) at 37 °C in a humidified atmosphere of 5% CO_2_. The culture medium contained 50% Dulbecco’s modified Eagle’s medium (DMEM) and 50% Ham’s F12 Nutrient Mixture (Merck, Darmstadt, Germany) supplemented with 1.5 mmol/L HEPES, 5% fetal bovine serum (FBS) (Biocenter, Budapest, Hungary), 1% insulin/transferrin/sodium selenite medium supplement, 5 ng/mL epidermal growth factor (EGF) and 1% penicillin/streptomycin (all purchased from Invitrogen, Thermo Fisher Scientific, Waltham, MA, USA). Cells were used between passages 42 and 45. The media were changed every second day.

### 2.3. Treatments of IPEC-J2 Cells with OTA, LPS and Flavonoids LUT and CHR

The stock solutions were freshly made with phenol red-free DMEM/F12 (Merck, Darmstadt, Germany). OTA was used at concentrations of 1 µM, 5 µM, 20 µM. CHR was applied at 1 µM, LUT was at 8.7 µM and LPS was used at 10 µg/mL. Cell cultures were exposed to the treatments for an incubation time of 24 h. After the treatment cell-free supernatants were collected for subsequent procedures such as IL-6/IL-8 and EC H_2_O_2_ determination. 2′-7′dichlorodihydrofluorescein diacetate (DCFH-DA) and 3-(4,5-dimethylthiazol-2-yl)-2,5-diphenyltetrazolium bromide (MTS) assays were completed with IPEC-J2 cells seeded on 24-well and 96-well plates (obtained from Costar Corning Inc., Corning, NY, USA).

### 2.4. Cell Viability Evaluation

Cytotoxicity was examined with MTS reagent (CellTiter96 Aqueous One Solution, Promega, Madison, WI, USA) in IPEC-J2 cells. This test measures the rate of metabolically active cells with the advantage over MTT that the solubilization step can be avoided.

IPEC-J2 cells were seeded on 96-well culture plates and allowed 24 h to reach confluence. OTA in the range of 1 µM to 20 µM, 10 µg/mL LPS, 8.7 µM LUT, 1 µM CHR solutions as well as their combinations were added to the cells using a multichannel pipette and were incubated for 24 h at 37 °C, 5% CO_2_. After the incubation time, the treatments were removed, and each well received 100 µL of fresh phenol red-free medium containing 20 µL of MTS solution. After an incubation time of 2 h at 37 °C, the absorbance values were measured at 490 nm using the SpectraMax iD3 microplate reader. MTS experiments were done four times for each treatment group except in the case of LPS administration upon which three parallels were carried out.

### 2.5. Detection of Changes in the Redox Status of IPEC-J2 Cells

Effects of *E. coli* LPS (10 µg/mL), OTA (in the concentration range of 1 µM and 20 µM), the flavonoids (1 µM CHR and 8.7 µM LUT) alone or in combinations were tested on the redox status of IPEC-J2 cells. All chemicals were dissolved in phenol-red free plain medium and were incubated with the cells for 24 h on 24-well plates. EC H_2_O_2_ production was monitored in IPEC-J2 cells by using the Amplex red hydrogen peroxide assay kit. After treatment, 50 µL of the cell-free supernatants was collected and was mixed with the Amplex red working solution according to the manufacturer’s instructions. The fluorescence intensity was measured with Victor X2 2030 fluorometer (excitation and emission wavelengths were 530 nm and 590 nm, respectively). Cells treated only with plain medium served as control. Amplex red experiments were repeated fourfold per each group.

To detect the amount of IC ROS, 10 µM DCFH-DA dye (Merck, Budapest, Hungary) was used. IC ROS can oxidize DCFH-DA to a detectable fluorescent product (DCF), therefore, elevated fluorescence values show an increased amount of IC ROS. The dye was added to the cells for 60 min, followed by rinsing with medium, scraping and centrifugation for 10 min (at 3000× *g*). A Victor X2 2030 fluorometer was used to determine the fluorescence of the samples (excitation wavelength: 480 nm, emission wavelength: 530 nm). DCF measurements were repeated three times per each group.

### 2.6. Determination of Proinflammatory Cytokine IL-6 and IL-8 Expression

The cell-free supernatants of IPEC-J2 exposed to LPS, OTA, different flavonoids and their combinations were collected after 24 h of incubation time. Porcine IL-6 and IL-8 sandwich ELISA kits were used to detect the changes in cytokines’ levels. The supernatants were treated according to the instructions of the manufacturer and measured by SpectraMax iD3 microplate reader at 450 nm. Three–three parallel experiments were accomplished during IL-6 and IL-8 determinations. 

### 2.7. Statistical Analysis

The statistical determination of the data was performed by using R Core Team (version of 2016). Differences between groups were estimated by one-way ANOVA using the post hoc Tukey test for multiple comparisons. *p* < 0.05, *p* < 0.01, and *p* < 0.001 indicate statistically significant differences.

## 3. Results

### 3.1. Cell Viability Assay on the IPEC-J2 Cell Line

**Figure 3:**
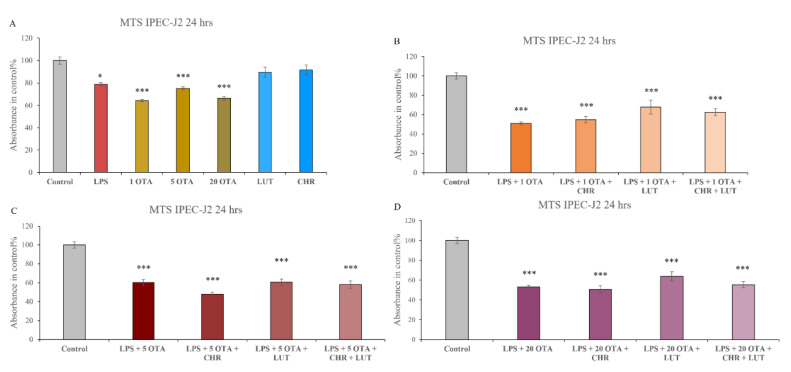
Cell viability determination. Changes in IPEC-J2 cell viabilities were detected after 24 h of administration of 10 µg/mL LPS, 1 µM OTA, 5 µM OTA, 20 µM OTA and in their combinations in the absence and in the presence of 1 µM CHR and 8.7 µM LUT (**A**–**D**). Data are shown as means of absorbances expressed in control % with standard errors of mean (SEM); n = 3–4 samples per group; * indicates *p <* 0.05, *** indicates *p <* 0.001 compared to controls.

After 24 h of incubation viability of IPEC-J2 cells was measured with MTS (Figure 3) on the IPEC-J2 cell line. CHR (1 µM) and LUT (8.7 µM) alone did not show any negative effect on the viability of cells. LPS (10 µg/mL) could reduce cell viability (*p =* 0.0486), and in combination with OTA (1 µM, 5 µM, 20 µM) a significant worsening of the viability can be seen (*p <* 0.001). OTA alone had shown to be capable of inducing cell death (1 µM, 5 µM, 20 µM *p <* 0.001 in each case). As seen in Figure 3, a significant reduction in cell viability can be detected in case of all the combinations of LPS + OTA + CHR (10 µg/mL + 1 µM/5 µM/20 µM + 1 µM) as well as LPS + OTA + CHR + LUT (10 µg/mL + 1 µM/5 µM/20 µM + 1 µM + 8.7 µM) and LPS + OTA + LUT (10 µg/mL + 1 µM/5 µM/20 µM + 8.7 µM) (*p <* 0.001). After our investigation, it can be stated that the significant cytotoxic effect of LPS, OTA and LPS + OTA could not be decreased by the application of flavonoids LUT and CHR.

### 3.2. Detection of EC Hydrogen-Peroxide Production

**Figure 4:**
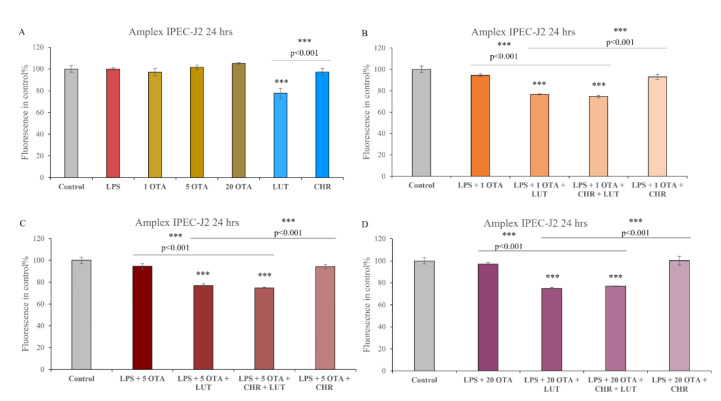
Relative fluorescence intensity in control% using Amplex red method. Changes in fluorescence intensities can be seen after 24 h of the combined treatment of cells with 1 µM OTA, 5 µM OTA, 20 µM OTA, 1 µM CHR, 8.7 µM LUT, 10 µg/mL LPS (**A**–**D**); data are shown as a means of relative fluorescence intensities with SEM; n = 4 samples per group; *** indicates *p <* 0.001, λ_exc_ = 530 nm, λ_em_ = 590 nm. Asterisks alone indicate significant differences between control and treated groups, asterisks with *p* values underlined show significant changes between the designated groups.

No significant elevation in H_2_O_2_ secretion could be seen in IPEC-J2 cells exposed to LPS (10 µg/mL) or to OTA (1 µM, 5 µM, 20 µM) or to their combination (*p >* 0.05). CHR at 1 µM did not show significantly lower H_2_O_2_ production; however, LUT (8.7 µM) could significantly decrease the peroxide levels (*p <* 0.001). A combination of LPS (10 µg/mL) + OTA (1 µM, 5 µM, 20 µM) with LUT (8.7 µM) or LUT + CHR (8.7 µM + 1 µM) has shown to be capable of reducing the EC H_2_O_2_ (*p <* 0.001). CHR alone however as an additive to LPS + OTA (1 µM, 5 µM, 20 µM) was not capable of decreasing the H_2_O_2_ level. Based on our results, after 24 h of incubation, LPS and OTA could not significantly elevate the EC H_2_O_2_ levels, but LUT alone could reduce the H_2_O_2_ level effectively. CHR alone was not able to affect the EC H_2_O_2_ level (Figure 4).

### 3.3. IC ROS Determination

**Figure 5:**
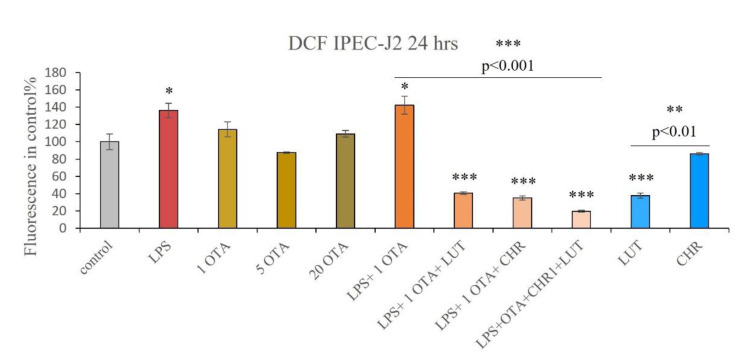
Relative fluorescence intensity in control% using DCFH-DA method. Measurement of IC ROS levels after 24 h treatment of IPEC-J2 cells with 1 µM OTA, 5 µM OTA, 20 µM OTA, 1 µM CHR, 8.7 µM LUT, 10 µg/mL LPS and their combinations; data are shown as means of relative fluorescence intensities with SEM; n = 3 samples per group, * indicates *p <* 0.05, ** indicates *p <* 0.01, *** indicates *p <* 0.001; λ_exc_ = 480 nm, λ_em_ = 530 nm. Asterisks alone indicate significant differences between control and treated groups, asterisks with *p* values underlined show significant changes between the designated groups.

OTA alone (1 µM, 5 µM, 20 µM) did not cause significantly higher IC ROS production in the IPEC-J2 cells (Figure 5). CHR (1 µM) alone did not seem to influence the IC redox status. Cells exposed to LPS (10 µg/mL) alone have significantly higher levels of ROS (*p =* 0.0443) similarly to those in IPEC-J2 cells challenged with the co-administration of LPS + OTA (10 µg/mL + 1 µM) (*p =* 0.0109). LUT (8.7 µM) alone, as well as CHR (1 µM) alone and their combination LUT + CHR (8.7 µM + 1 µM), have shown to be capable of lowering IC ROS induced by LPS + OTA (10 µg/mL + 1 µM) (all *p <* 0.001). LUT (8.7 µM) could significantly decrease the oxidative stress within the cells (*p <* 0.001). Based on our 24 h findings, it can be stated that LPS alone, as well as LPS + OTA, resulted in a higher level of ROS in the cells which can be successfully prevented by LUT and CHR alone as well as by their combination.

### 3.4. The Changes in IL-6 and IL-8 Levels after Exposure to OTA, LPS and the Selected Flavonoids in IPEC-J2 Cells

**Figure 6:**
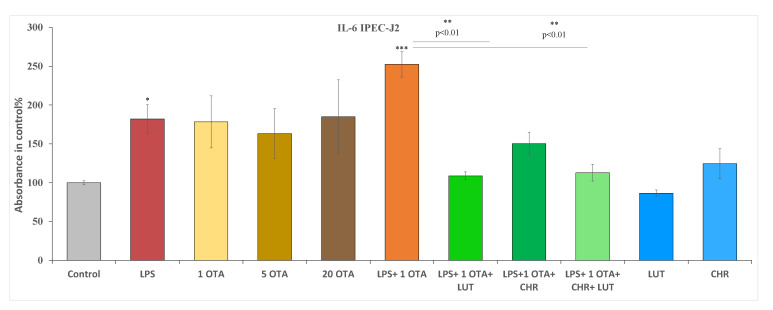
Measurements of interleukin-6 levels after 24 h of treatment of IPEC-J2 cells with 1 µM OTA, 5 µM OTA, 20 µM OTA, 1 µM CHR, 8.7 µM LUT, 10 µg/mL LPS and their combinations; data are shown as means of absorbance values with SEM; n = 3 samples per group; * is *p <* 0.05, ** and *** indicates *p <* 0.01 and *p <* 0.001, respectively. Asterisks alone indicate significant differences between control and treated groups, asterisks with *p* values underlined show significant changes between the designated groups.

**Figure 7:**
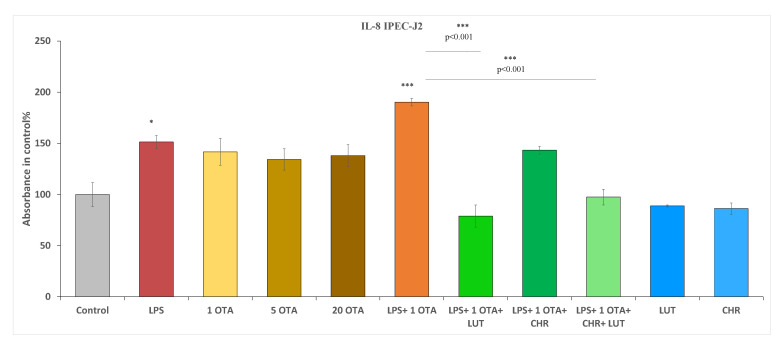
Measurements of interleukin-8 levels after 24 h treatment of IPEC-J2 cells with 1 µM OTA, 5 µM OTA, 20 µM OTA, 1 µM CHR, 8.7 µM LUT, 10 µg/mL LPS and their combinations; data are shown as means with SEM; n = 3 samples per group, * indicates *p <* 0.05, *** indicates *p <* 0.001. Asterisks alone indicate significant differences between control and treated groups, asterisks with *p* values underlined show significant changes between the designated groups.

Different concentrations of OTA (1 µM, 5 µM and 20 µM) were used in cells for IL-6/IL-8 examinations. Additionally, 10 µg/mL LPS alone, LPS + OTA combination (10 µg/mL + 1 µM), flavonoids 1 µM CHR, 8.7 µM LUT alone and these in combinations with LPS + OTA were used to see their inflammatory effects (Figure 6 and Figure 7). Based on our 24 h findings, IL- 6 production was not increased in IPEC-J2 cells exposed to OTA at 1 µM, 5 µM and 20 µM compared to untreated controls. LUT and CHR did not influence the basal IL-6 secretion of the cells. However, when LPS (10 µg/mL) or LPS + OTA combination was used (10 µg/mL LPS + 1 µM OTA), significant increase in IL-6 levels was detected (*p* = 0.04431 and *p* < 0.001, respectively). LUT (8.7 µM) and LUT + CHR (8.7 µM + 1 µM CHR) could efficiently reduce IL-6 production to the control level when these flavonoids were administered simultaneously with LPS + OTA for 24 h (both *p* < 0.001). It was also found that LUT and LUT + CHR administration reduced IL-6 oversecretion in a similar manner (*p* = 0.0717) in IPEC-J2 cells exposed to OTA and LPS (1 µM + 10 µg/mL) together (Figure 6).

LPS at 10 µg/mL could elevate proinflammatory IL-8 levels significantly (*p* = 0.0489) after 24 h of treatment. OTA alone even at very high concentration (at 20 µM) could not promote IL-8-mediated inflammation (*p* > 0.05). Beneficial effects could be seen with the application of LUT and CHR at 8.7 µM and 1 µM when they were administered simultaneously; however, LUT alone could suppress LPS + OTA-caused IL-8 production in a very similar manner as it could be observed in the case of administration for LUT + CHR combination (both *p* < 0.001). No significant changes in IL-8 secretion were found between LPS + OTA + LUT and LPS + OTA + CHR + LUT; thus, CHR alone did not exert a beneficial effect (*p* = 0.0807) on decreasing upregulated IL-8 levels (Figure 7).

In accordance with our 24 h results treatment of IPEC-J2 cells with OTA and LPS led to a significant elevation of proinflammatory IL-6 and IL-8 cytokine productions which were successfully alleviated by LUT and LUT + CHR. CHR addition did not improve the efficacy of LUT in restoring LPS + OTA-facilitated inflammatory processes. 

## 4. Discussion

Reducing the development of antibiotic resistance is one of the most important challenges in human and veterinary medicine today. Numerous European Union (EU) guidelines were issued on prudent antibiotic usage, and an important goal of the 2019/6 EU regulation on veterinary medicinal products—to be entered into force in 2022—is to further regulate the antibiotic usage by practicing veterinarians [29]. To avoid the immunosuppressive effects of environmental stress in industrial livestock production, farmers should strive to use good husbandry practices and feeds and could also apply a range of natural medicinal products to improve animal health. A major advantage of the alternative products is that they do not require the setting of a maximum residue limit (MRL) and therefore do not have to be subject to a withdrawal period. When considering certain flavonoids as medicines to treat disease and prevent stress, it is important to note that although these flavonoids are present in most food sources and have beneficial effects on human health, little is known about how exactly they work and what the effects of ingesting these compounds are on animals [7].

Several pathological factors in the intestinal tract can lead to the development of dysbiosis. Genetic factors, diet, antibiotic therapy, and environmental stressors can all play a role in upsetting the microbial balance [30]. Pathogenic bacterial overgrowth leads to oxidative stress, inflammation of the intestinal epithelial cells, reduced intestinal barrier function and ultimately increased levels of antigens and toxins [31]. An inadequate redox status can lead to detrimental changes in membrane lipids, structural proteins and DNA, affecting cell viability, cell division and, thus, organ function. All these negative effects can be exacerbated in the presence of other environmental stressors.

The presence of Gram-negative enteric pathogens and other environmental stressors, such as mycotoxins, could result in reduced enterocyte viability. Flavonoids may have a role in preventing cell death, but the concentration and duration of treatment at which these compounds can be safely administered should also be investigated. LUT has previously been shown not to reduce enterocyte viability when applied to IPEC-J2 cells at a concentration of 100 µM for 24 h [32]. In the present study, similar results were obtained, i.e., the addition of LUT at 8.7 µM did not affect the viability of IPEC-J2 cells. To our best knowledge, the effect of CHR on viability in IPEC-J2 cells has not been investigated earlier. We have shown that CHR at a concentration of 1 µM does not negatively affect the viability of IPEC-J2 cells after 24 h of treatment and is therefore safe. LPS of *E. coli* origin reduced significantly the number of living enterocytes. In addition, a significant cytotoxic effect was also observed for OTA, similarly to the study by Wang et al. [18]. CHR and LUT were not effective in preventing cell death caused by a 24-hour-lasting combined exposure of LPS (10 µg/mL) and OTA (1, 5, 20 µM). In Caco-2 intestinal cells, a concentration-dependent effect of OTA was described, with cytotoxic effects appearing above a concentration of 40 µM [33]. However, Caco-2 is a cancerous cell line with a presumably higher resistance compared to IPEC-J2. Interestingly, the presence of resveratrol polyphenol did not decrease but increased the toxicity of OTA in the above-mentioned study.

Both LUT and CHR are potent antioxidants, with the hydroxyl groups on their rings and the double bond is found at 2–3 positions on the C ring [34]. For LUT, the antioxidant effect is further enhanced by the catechol structure on the B ring. LUT was more effective than vitamin C in reducing ROS and increasing the activity of superoxide dismutase and catalase in human leukocytes and rat blood, thus, preventing lipid peroxidation [35]. Its antioxidant effect has also been investigated in IPEC-J2 cells. Kovács et al. showed [32] that the increase in ROS levels induced by *Salmonella enterica*, and various *E. coli* endotoxins were significantly reduced by the presence of LUT, and the effect was independent of concentration (25-100 µM). This agrees with our results, where LUT, even at a lower concentration (8.7 µM) was successful in reducing LPS and OTA combination-induced oxidative responses. The effect of CHR was similar to that of LUT, we were the first to show that the presence of CHR contributes to the attenuation of *E. coli* LPS and OTA-triggered oxidative stress in IPEC-J2 cells.

The oxidative stress-inducing effect of OTA on IPEC-J2 cells has not yet been investigated but has been studied in other in vitro cultures. Vero cells treated with 10 µM OTA for different time intervals resulted in elevated IC ROS levels [36]. It was also demonstrated, that LUT alleviates OTA-induced oxidative stress by regulating nuclear factor erythroid 2–related factor 2 (Nrf2) and hypoxia-inducible factor-1 α (HIF-1α) pathways in NRK-52E rat kidney cells [37]. The oxidative stress-inducing impact of OTA was investigated by Wang et al. [28] in IPEC-J2 cells using the DCFH-DA method and a concentration-dependent effect was described; however, the treatment was continued for 12 h. It was found in our study that 24 h of treatment of OTA did not increase IC ROS levels at the concentrations used, but it is possible that this phenomenon is due to a compensatory effect of viable cell number reduction caused by the toxic effect of the mycotoxin. The Amplex red method, in contrast to DCF, is only suitable for the detection of H_2_O_2_, whereas the latter is a non-selective ROS detecting method. In IPEC-J2 cells, LPS of various origins could not increase H_2_O_2_ levels [38] using Amplex red method which is in good agreement with our present findings. Other mycotoxins such as DON and T2 toxin and their combination could increase EC H_2_O_2_ in IPEC-J2 cells, which returned to control levels in the presence of rosmarinic acid [39].

A large part of the outer cell wall of Gram-negative bacteria is made up of LPS, which helps the bacteria to bind to host cells and activates the innate immune system and immune response by binding to toll-like receptor 4 (TLR4) [40]. Gram-negative bacteria in contact with intestinal epithelial cells can increase ROS production by enterocytes, which can lead to apoptosis of infected cells [40], with the consequent vulnerability of the cell layer integrity [41]. ROS increase the levels of certain cytokines in blood plasma and intestinal mucosa [42]. The nuclear factor kappa B (NF-κB) signaling pathway plays an important role in the regulation of cytokine production and activation of the innate immune system. NF-κB facilitates the recognition of immune-stimulating molecules and influences the stimulating or inhibiting of the synthesis of inflammatory cytokines [40]. The activation of the TLR-4-signaling pathway was described after the OTA treatment resulting in elevated mRNA expression and protein levels of TLR4 Myd88 and p-p65 in duck liver samples. OTA administration also promoted the mRNA expression and secretion of inflammatory cytokines, including IL-6 [43]. OTA was reported to increase the secretion of pro-inflammatory cytokines such as IL-6 in isolated jejunal tissue culture and affect immune function in the intestine [17]. The study of the combined effect of OTA and LPS, modelling the complexity of environmental factors and their effects on immunological parameters, has not been done before. In our study, applied LPS-induced changes in IL-6 and IL-8 levels after 24 h. OTA treatments were not associated with increases in these cytokine levels. It is noteworthy, however, that co-administration of OTA and LPS induced significant increases in both cytokines tested, thus, modelling multiple exposures to environmental stresses. Moreover, the application of LUT decreased the levels of IL-6 and IL-8 in the IPEC-J2 cell culture. 

IPEC-J2 is a morphologically and functionally more differentiated cell line compared to other porcine intestinal cells such as IPEC-1 and IPI-2I thus it is a preferential tool for in vitro studies; however, even non-tumorigenic cell models have their own limitations due to the fact that they fail to show the exact behaviors of the cells in an organism.

## 5. Conclusions

In conclusion, LUT and CHR could counteract the combined damaging effects of *E. coli* LPS and OTA, but further in vivo tests are needed to understand the background of the complex effects. The adverse effects of Gram-negative pathogenic bacteria and OTA on the digestive tract in pigs are significant. Intestinal cells are the first cells to be exposed to harmful agents, often at higher concentrations than other tissues. The presence of natural substances such as flavonoids in feed can compensate for the effects of these environmental stressors and contribute to reducing the use of antibiotics, thereby decreasing the spread of antimicrobial resistance and the likelihood of harmful factors entering the food chain. Currently, there have been no commercial feed additives available for swine containing LUT or CHR, but further in vivo investigations can clarify optimal dosage protocols for their future usage in veterinary practice.

## Data Availability

All data that supports above-detailed findings can be obtained from the corresponding author upon request.
